# Supplementary material for: The Evolution and Characterization of the RNA Interference Pathways in Lophotrochozoa
Source: Genome Biol Evol. 2024 May 7;16(5):evae098. doi: 10.1093/gbe/evae098 (PMC11114477; doi:10.1093/gbe/evae098)
Supplement: evae098_Supplementary_Data [file evae098_supplementary_data.zip › Supplementary_materials.zip!/selected_miRNAs/NoGe_selected_miRNAs/novel_miRNA_families_pdf_files/NMRB01001254.1_Notospermus_geniculatus_strain_Ushimado_scaffold1254,_whole_genome_shotgun_sequence_26720.pdf]

Provisional ID	:	NMRB01001254.1_Notospermus_geniculatus_strain_Ushimado_scaffold1254,_whole_genome_shotgun_sequence_26720
Score total	:	88862.4
Score for star read(s)	:	3.9
Score for read counts	:	88854
Score for mfe	:	1.5
Score for randfold	:	0
Score for cons. seed	:	3
Total read count	:	174295
Mature read count	:	174095
Loop read count	:	0
Star read count	:	200

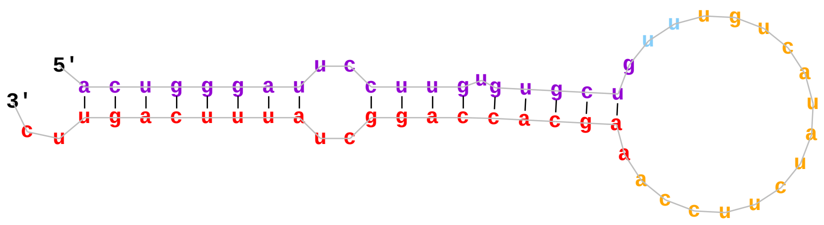

Genome coordinates: NMRB01001254.1 23242-23301

pre-miRNA sequence:
ACTGGGATTCCTTGTGTGCTGACTGGGATTCCTTGTGTGCTGTTTGTCATATCTTCCAAAGCACCAGGCTATTTCAGTTC

Mature sequence: AAGCACCAGGCTATTTCAGTTC

BLAST results for the pre-miRNA sequence against the *Lineus longissimus* genome assembly:

**Scaffold**	**Start**	**Stop**	**Identity**	**e-value**
OU342992.1 1	19328788	19328846	88.136	4.73e-12

```
Nge_novel_miRNA     ACTGGGATTCCTTGTGTGCTGTTTGTCATATCTTCCAAAGCACCAGGCTATTTCAGTTC
Llo_genome          ACTGGGATACCATGTGTGCTATTTGTGATTACTGCCAAAGCACCAGGCTATTTCAGTTC
```
